# Exploring the Glycolytic Mechanisms in “Driver Gene-Negative” Lung Adenocarcinoma (LUAD): A Single-Cell RNA Sequencing Approach to Identify the MIF-HIF-1α Axis

**DOI:** 10.7150/jca.119149

**Published:** 2025-10-10

**Authors:** Hao-Shuai Yang, Yuan-Hao Li, Qi Chen, Hong-He Luo, Qi-Duo Yu, Yu Han, Weijie Zhu, Jin Zhang, Chao-Yang Liang

**Affiliations:** 1 Department of Thoracic Surgery, China-Japan Friendship Hospital, Beijing 100029, P.R. China.; 2 Department of Thoracic Surgery, The First Affiliated Hospital of Sun Yat-sen University, Guangzhou 510080, P.R. China.

**Keywords:** lung adenocarcinoma, driver gene-negative, glycolysis, MIF, HIF-1α, multi-omics analysis

## Abstract

**Background**: Patients with "driver gene-negative" LUAD lack effective targeted therapies. This study aimed to elucidate the role of the glycolysis pathway in driver gene-negative LUAD to identify key genes and potential therapeutic targets.

**Methods**: Bulk RNA sequencing data from 49 patients with driver gene-negative LUAD were analyzed. The driver gene-negative status of patients was confirmed by immunoblotting. Gene set enrichment analysis (GSEA) was conducted on six hallmark pathways related to glycolysis. Additionally, key genes were identified and a risk score model was constructed. Finally, single-cell RNA sequencing data were processed using the Seurat package for data cleaning, dimensionality reduction clustering, and cell type identification.

**Results**: GSEA analysis revealed significant enrichment of the glycolysis pathway in driver gene-negative LUAD. Differential expression analysis identified 144 genes associated with the glycolysis pathway. Six glycolysis-related genes (ANKZF1, GPR87, KIF2A, LCT, MIF, SDHC) were identified associated with poor prognosis. Single-cell sequencing analysis validated the key role of MIF in the glycolysis process and revealed a positive feedback regulatory axis between MIF and HIF-1α, which may promoting glycolysis and malignant transformation.

**Conclusion**: This study elucidated glucose metabolic reprogramming mechanisms and highlighted the MIF-HIF-1α axis as a promising therapeutic target in "driver gene-negative" LUAD, which may offer new avenues for improving outcomes, particularly those lacking conventional targeted therapy options.

## Introduction

As is well documented, lung cancer is one of the most prevalent cancers and the leading cause of cancer-related deaths worldwide^1^. Lung adenocarcinoma (LUAD) is the most common histological type of lung cancer, accounting for over 40% of the total cases^2^. In recent years, the exploration of tumor driver genes and the rapid development of molecular detection technology has led to the identification of a series of driver genes in the field of LUAD, such as EGFR, KRAS, BRAF, HER2, MET, RET, ROS1 and ALK^3^. Targeted drugs have been developed for some of these genes, significantly improving the survival rates of patients harboring driver gene mutations^4^. However, a subset of LUAD patients lacks detectably mutational sites corresponding to targeted drugs, referred to as "driver gene-negative" lung adenocarcinoma patients, which accounts for approximately 22%-55% of all LUAD cases^5^. These patients have relatively limited treatment options, highlighting the urgent need to explore new therapeutic methods to improve their prognosis. Therefore, a multi-omics cohort of patients with LUAD negative for major driver gene mutations (EGFR, KRAS, BRAF, HER2, MET, ALK, RET, and ROS1)^6^ was established to comprehensively explore the pathogenic molecular mechanisms underlying this subtype and identifying effective therapeutic targets for this population.

In 1923, Otto Warburg^7^ described that tumor tissue slices consumed high levels of glucose and synthesized lactate under aerobic conditions, a phenomenon known as the "Warburg effect". Other forms of metabolic reprogramming have been discovered on this basis and have been hypothesized to play a vital role in tumor proliferation and metastasis^8^, ^9^. While many studies have reported the role of glycolysis in the occurrence and development of lung cancer^10^, abnormalities in the glycolysis pathway seem to play a more critical role in driver gene negative LUAD. Our previous work demonstrated that glycolysis is the most discriminative metabolic features distinguishing driver gene-negative LUAD from adjacent tissues^11^, ^12^. However, the mechanism underlying the role of glucose metabolism reprogramming in driver gene-negative lung adenocarcinoma remains elusive.

Therefore, this study utilized bulk and single-cell RNA sequencing sequencing to further explore glycometabolism-related pathways to identify key genes and potential mechanisms that affect the development and progression of driver gene-negative lung adenocarcinoma, to determine cell types involved at the cellular level and to use cell line models to conduct preliminary validation.

## Methods

### Patient selection and data enrollment

The bulk-RNA sequencing data of 49 patients with driver gene-negative LUAD was retrieved from a previously published cohort^11^, ^13^, ^14^ comprising 626 formalin-fixed, paraffin-embedded (FFPE) tumor and healthy tissue samples between September 2003 and June 2015. No patients underwent antitumor therapy prior to biopsy sampling. Immunohistochemical staining and immunoblotting assays were performed on FFPE tissues to confirm driver gene-negative status. Driver gene-negative status was defined as the absence of mutations in ALK, EGFR, HER2, KRAS, MET, BRAF, ROS1, and RET, which precluded the use of current mainstream targeted therapies.

**Figure 1:**
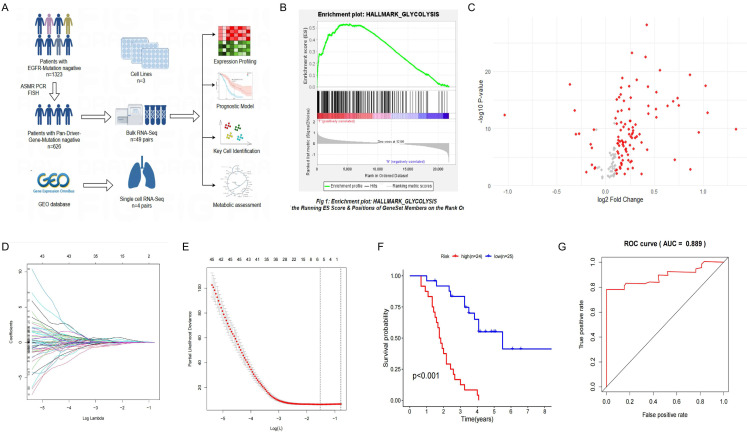
Construction of a Glycolysis-Related Prognostic Model for Patients with "Driver Gene-Negative" Lung Adenocarcinoma. (A) Sample type and main analysis process. (B) Glycolysis pathways are significantly enriched in tumor tissues of "driver gene-negative" lung adenocarcinoma. (C) Differential gene expression between "driver gene-negative" lung adenocarcinoma tumor tissues and adjacent normal tissues. (D) LASSO coefficient pathway diagram for differentially expressed glycolysis-related genes. (E) LASSO regression cross-validation curve. (F) Survival analysis of high and low-risk groups in the glycolysis-related prognostic model. (G) ROC curve for the glycolysis-related predictive model.

Single-cell RNA sequencing data were acquired from GSE131907^15^, which included tumors and adjacent tissues of four driver gene-negative patients. The workflow for sample collection, analysis, and processing is illustrated in Figure 1A.

### Gene set enrichment analysis (GSEA) of glucose metabolism-related pathways

Six glucose metabolism-related hallmark pathways were identified in the Molecular Signatures Database (MSigDB)^16^, namely glycolysis, oxidative phosphorylation, oxidative respiratory chain assembly, gluconeogenesis, pentose phosphate pathway, and glycogen synthesis and decomposition. Gene set enrichment analysis was conducted using GSEA software (v.4.3.2) to compare these pathways between tumor and normal groups. *p*<0.05 was considered statistically significant.

### Identification and prediction model construction of key genes

Differential expression analysis was performed between tumor and healthy tissues. Differentially expressed genes (DEGs) were identified based on the thresholds adj.*p*<0.05 and |log2 (fold-change)| > 0.5. Next, the least absolute shrinkage and selection operator (LASSO) regression analysis was performed on DEGs between the tumor and adjacent control groups to confirm the optimal lambda value and identify key genes. Utilizing these pivotal genes, a risk score model was constructed for patients with driver gene-negative lung adenocarcinoma. The risk score was calculated as follows:



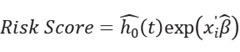



Model performance was assessed using the ROC curve. The median risk score was used to stratify patients into high-risk and low-risk groups and visualized using the "survival", "glmnet", "pbapply", and "survivalROC" R packages.

### Single-cell data processing and cell type determination

The dataset was curated by excluding cells with nFeature_RNA counts greater than 200 or less than 5000. Additionally, to ensure the integrity of the analysis, cells exhibiting mitochondrial gene expression exceeding 15% were systematically excluded. Further analysis was performed using the R package "Seurat" after cleaning and quality control of raw data. The FindClusters function was used to identify functional characteristics of various cell clusters, with a resolution of 0.4 and PC=25 for cell clustering based on the expression profiles of each cell cluster.

The marker genes for each cluster were identified using the "FindMarkers" function. The cell types in each cluster were annotated according to human lung cell marker genes established in previous studies^17^. Epithelial cells are recognized as cells of origin for lung adenocarcinoma. To further explore the roles of distinct epithelial cell subpopulations in the progression of driver gene-negative lung adenocarcinoma, EPCAM+ cells were categorized as epithelial, and subcluster analysis was performed.

### Copy number variation (CNV) analysis

CNVs analysis was conducted to determine the malignancy of epithelial cells. To delineate cellular trajectories, high CNV-score epithelial cells were isolated from the squamous epithelium and classified as malignant epithelial cells. Following this, the Monocle2 algorithm was used, leveraging a gene-cell matrix derived from a scaled Unique Molecular Identifier (UMI) count dataset within the Seurat framework as the input. The analysis was conducted using default settings to predict cellular developmental paths using the R package "InferCNV".

### Gene set enrichment analysis with pathway activity estimation

Gene set enrichment analysis (GSEA) was conducted using 50 hallmark pathways from the Molecular Signatures Database (MSigDB) to evaluate their activity levels across various cell types. Additionally, Gene Set Variation Analysis (GSVA) was performed on each cell to estimate the enrichment scores for each pathway within individual cells. Differences between activity scores were used to quantify differential pathway activity among distinct cell subtypes.

### Gene Ontology (GO) and Kyoto Encyclopedia of Genes and Genomes (KEGG) pathway enrichment analysis

Enrichment analysis, including Gene Ontology (GO) and Kyoto Encyclopedia of Genes and Genomes (KEGG) enrichment analyses, were performed using R packages "edgeR", "org.Hs.eg.db", "enrichplot", and "ggplot2" to elucidate the different biological processes and pathways between the groups. *p*<0.05 was considered statistically significant.

### Cell line and cell transfection

Based on previous studies on "driver gene-negative" lung cancers^18^, we selected two lung cancer cell lines (H1703 and Calu-3 cells) as positive controls. The lung cancer cell lines and normal human alveolar epithelial type II (AT2) cells were sourced from the American Type Culture Collection and cultured in Dulbecco's Modified Eagle medium (DMEM; Thermo Fisher Scientific). MIF plasmids were purchased from Synechuang Bio. Briefly, AT2 cells were transfected with 5 μg plasmid using lipofectamine 3000 and lipofectamine 2000 reagents (Invitrogen). ISO-1 is a known antagonist of MIF that exerts its effect by inhibiting the D-dopachrome tautomerase activity of MIF. In the present study, the activity of MIF was inhibited using 100 μM ISO-1. Dimethyloxalylglycine (DMOG) is a small molecule compound that stabilizes HIF-1α by inhibiting the activity of prolyl hydroxylase. Herein, the function of HIF-1α was stabilized by the addition of 100 μM DMOG.

### [3H]-2DG method for detecting glucose uptake

The glucose uptake efficiency of cells can be assessed by measuring their uptake of [3H]-2DG. Cells were starved for 24 hours and then incubated in a DMEM culture medium supplemented with 37 kBq/mL [3H]-2DG (containing 1 mg/mL glucose) for an additional 24 hours. Next, cells were digested with trypsin, a small portion was retained for counting, and the remaining cells were centrifuged and lysed with 0.5 mol/L NaOH for 15 minutes. The cell lysate was neutralized with an equal volume of 0.5 mol/L hydrochloric acid. Finally, a liquid scintillation counter (HIDEX300SL, Finland) was used to measure the disintegrations per minute (DPM) of the cell lysate, whilst [3H]-2DG uptake was calculated using the following formula: [3H]-2DG uptake = (Experimental group DPM - Blank control group DPM)/(Control group DPM - Blank control group DPM).

### Microplate assay for lactate dehydrogenase (LDH) activity

The LDH activity was determined following the Lactate Dehydrogenase Activity Assay Kit (Sigma-Aldrich, MAK066). A total of 1×10^6^ cells was collected from each group, following which 100 μL of cell lysis solution was added. Next, the mixture was incubated on ice for 10 minutes, then centrifuged at 13,000g for 10 minutes to discard debris. The supernatant was collected thereafter. Then, lactate solution, 1× INT solution, and enzyme solution were mixed in equal volumes to prepare the working solution. Subsequently, 50 μL of the working solution was added to a 96-well plate and incubated at room temperature in the dark for 30 minutes. Absorbance was measured at 490 nm using a microplate reader. Relative LDH activity was calculated as follows: Relative LDH activity = (absorbance of the sample well - absorbance of the background blank control well)/(absorbance of the control group well - absorbance of the standard blank well).

### Microplate assay for lactate levels

Lactate levels were determined following the Lactate Assay Kit (Sigma-Aldrich, MAK064). Cells from each group were seeded at a density of 1×10^5^ cells per well in a 6-well plate and cultured for 12 hours, following which the medium was replaced with 1 mL of serum-free medium per well and incubated for an additional 24 hours. The cell culture medium was then collected and centrifuged at 13,000g for 10 minutes to remove impurities. A mixture of 20 μL of the sample, 26 μL of lactate assay buffer, 2 μL of lactate enzyme mixture, and 2 μL of lactate probe was prepared and incubated at room temperature for 30 minutes. Lastly, the absorbance of the sample was measured at 570 nm using a microplate reader.

### Assessment of cellular proliferation

To evaluate cell proliferation, Cell Counting Kit-8 (CCK-8) was used following the protocols provided by the manufacturer. An aliquot of approximately 100 μL of the cell suspension was carefully pipetted into each well of a 96-well microplate. Subsequently, the CCK-8 reagent and 10 μL of a detection reagent were introduced into each well. Following this, the optical density (OD) of each well was determined at a wavelength of 450 nm using a microplate spectrophotometer.

### Statistical analysis

Statistical analyses were conducted using R software (version 4.3.1, sourced from https://www.r-project.org). Depending on the distribution of the metric data, values were expressed as mean ± standard deviation for continuous variables and as frequencies (percentages) for categorical data. Kaplan-Meier survival curves were plotted to evaluate differences in survival outcomes between the groups. Wilcoxon rank-sum test and one-way ANOVA were used to compare differences between groups. All tests were two-sided, and *p*<0.05 was considered statistically significant.

## Results

### The glycolysis pathway is highly enriched in driver gene-negative LUAD

GSEA enrichment analysis on six glucose metabolism-related pathways from MSigDB in driver gene-negative lung adenocarcinoma identified glycolysis as the only pathway with a significant difference between tumor and healthy tissues, with the highest enrichment in driver gene-negative LUAD (Figure 1B). The results of other pathways are illustrated in Supplementary Figure 1. Differential expression analysis of glycolysis-related genes between the tumor and adjacent control groups yielded 144 DEGs (Supplementary Table 1), as displayed in Figure 1B, indicating that differences in glycolysis processes may be associated with the development of driver gene-negative lung adenocarcinoma.

### The model established based on the glycolysis pathway can effectively predict patient prognosis

After performing a differential analysis of glycolysis gene expression levels between the tumor and adjacent control groups, LASSO regression analysis was used to further screen potential feature genes from DEGs. By determining the optimal λ value using glmnet (Figure 1C, D), six glycolysis-related genes (ANKZF1, GPR87, KIF2A, LCT, MIF, SDHC) were identified that are associated with poor prognosis in patients with driver gene-negative LUAD. The regression coefficients for each gene are listed in Supplementary Table 2. To further validate the significance of key genes, a survival prognostic model was established (Figure 1E), and a ROC curve was generated to validate the effectiveness of the prognostic model (Figure 1F).

### Single-cell sequencing confirmed that MIF is a key gene involved in the glycolysis of driver gene-negative lung adenocarcinoma

**Figure 2:**
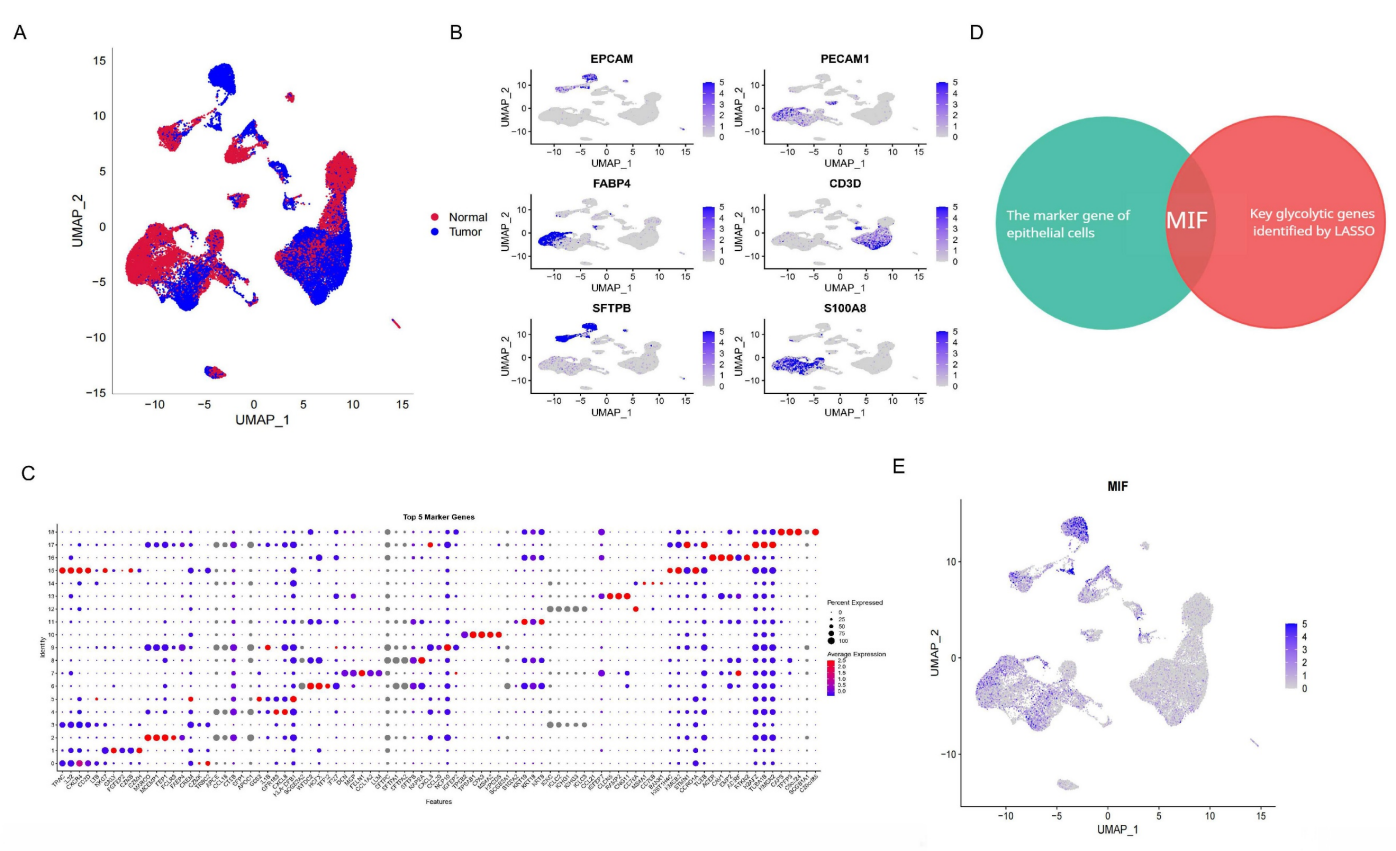
Single-Cell Sequencing Dimensionality Reduction and Clustering of "Driver Gene-Negative" Lung Adenocarcinoma. (A) Single-cell data from "driver gene-negative" lung adenocarcinoma, grouped by tumor and normal tissues after dimensionality reduction. (B) Expression of marker genes for various cell types; (C) Identification and expression of marker genes in each cluster. (D) MIF is the only gene intersecting between epithelial cell marker genes and glycolysis-related genes in "driver gene-negative" lung adenocarcinoma. (E) Expression of MIF at the single-cell level.

Single-cell sequencing data from driver gene-negative lung adenocarcinoma were used to further investigate the mechanisms underlying glycolysis-related genes. The results of data cleaning and identification of highly variable genes are depicted in Supplementary Figures 2A and B. After dimension reduction clustering of single-cell data derived from tumor and adjacent tissues from three patients, all cells were subdivided into 19 clusters, and the dimension reduction results were stratified by tissue origin, as shown in Figure 2A and Supplementary Figure 2C. Subsequently, cell annotation was performed using classic marker genes, namely EPCAM (epithelial cell), PECAM1 (endothelial cell), FABP4 (alveolar macrophages), CD3D (T cell), SFTPB (AT2 cell), and S100A8 (myeloid cell), with gene expressions levels delineated in Figure 2B and marker genes in each cluster shown in Figure 2C. Clusters 6/8/11 were identified as epithelial cells containing 717 marker genes. Previous studies have demonstrated that alveolar epithelial cells are the origin cells of lung adenocarcinoma^34^. Interestingly, intersecting the previously identified glycolysis-related genes with the marker genes of alveolar epithelial cells revealed that MIF can simultaneously serve as a differential gene in the glycolysis pathway and a marker gene for alveolar epithelial cells (Figure 2D), indicating that MIF is a key gene involved in regulating glycolysis in driver gene-negative lung adenocarcinoma. Its expression at the single-cell level is presented in Figure 2E.

### MIF+AT2 cells have higher glycolysis levels and a higher degree of malignancy

**Figure 3:**
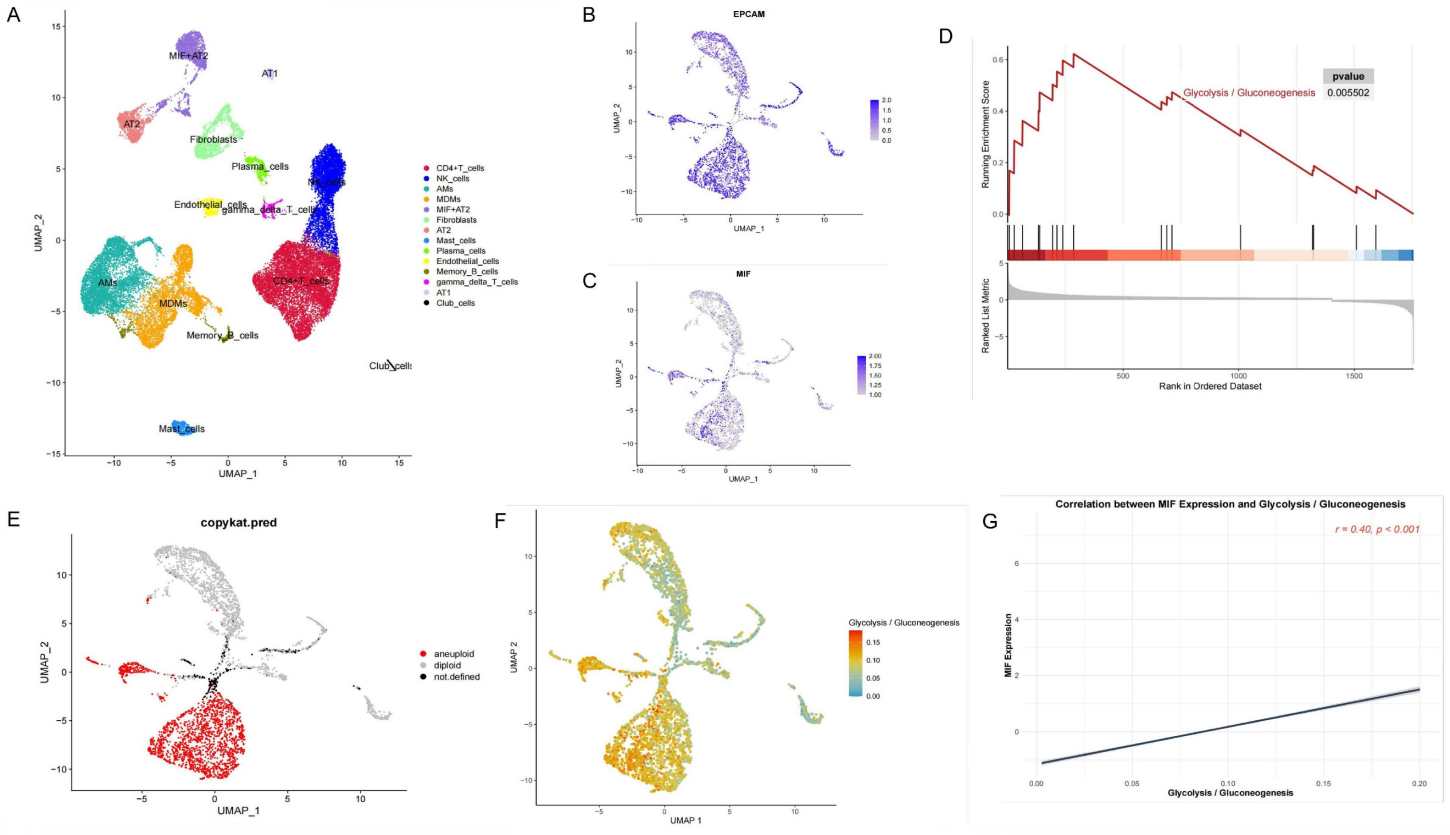
Analysis of Epithelial Cells in "Driver Gene-Negative" Lung Adenocarcinoma. (A) Cell annotation from single-cell sequencing data of "driver gene-negative" lung adenocarcinoma patients; (B) Expression of marker genes in the re-clustering analysis of epithelial cells; (C) Expression of MIF in epithelial cells; (D) Single cell GSEA analysis reveals significant enrichment of glycolytic pathways in MIF+AT2 cells; (E) CopyKAT analysis in epithelial cells; (F) Distribution of glycolytic active cells among epithelial cells; (G) Relationship between MIF expression and glycolytic levels.

Annotation of single-cell data based on the expression of marker genes yielded 14 cell types (Figure 3A). Among them, AT2 cells were further subclassified into MIF+AT2 cell subsets based on the expression level of MIF. GSEA comparing MIF+AT2 cell subsets with other AT2 cell subsets revealed that the glycolysis pathway was significantly more active in MIF+AT2 cell subsets (Figure 3B and Supplementary Figure 2D). To further explore the expression of MIF in various epithelial cell subsets, a re-clustering analysis of epithelial cell subsets was performed (Supplementary Figure 2E), and the expression of EPCAM and MIF in epithelial cell subpopulations is delineated in Figures 3C and D. Meanwhile, comparison with the results of cell malignancy inferred based on chromosome ploidy revealed a high degree of consistency between the distribution of epithelial cells with high MIF expression and aneuploid nuclear type, suggesting a correlation between MIF expression and cell malignancy (Figure 3E). Furthermore, We evaluated the correlation between MIF expression and various metabolic pathways and found that oxidative phosphorylation, glycolysis, and other O-glycan biosynthesis were more than 0.3 correlated with MIF expression, suggesting a close relationship between MIF and glucose metabolism (Supplementary Figure 2F). The results of glycolysis pathway scoring, based on the AUcell algorithm, uncovered a significant positive correlation between the glycolysis activity of epithelial cells and their MIF expression levels (Figures 3F and G). At the overall level, driver gene negative LUAD also showed higher levels of glycolysis compared to adjacent tissues (Supplementary Figure 2G).

### HIF-1α pathway is a key regulatory pathway for MIF

**Figure 4:**
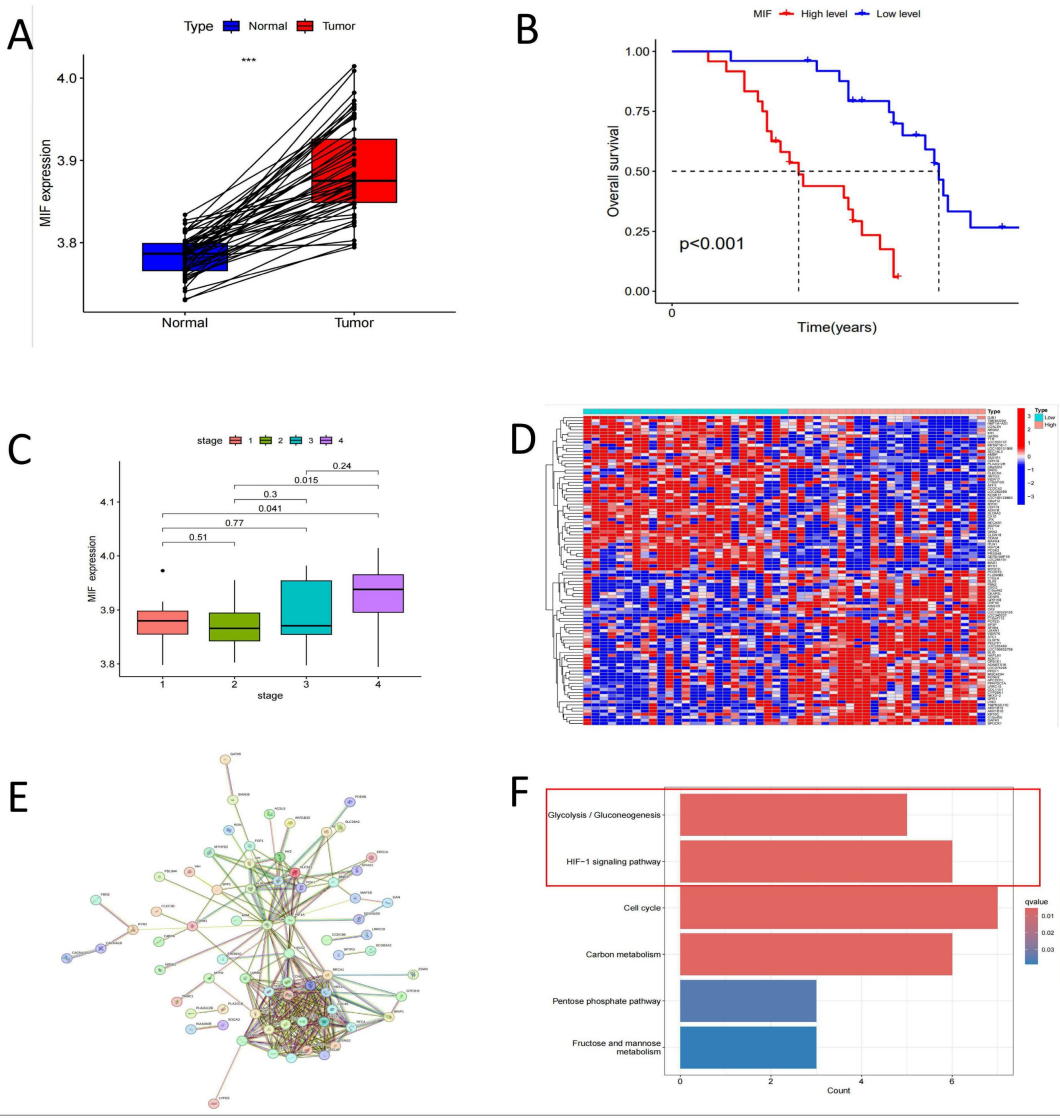
Correlation Analysis of MIF with Clinical Characteristics in "Driver Gene-Negative" Lung Adenocarcinoma. (A) Comparison of MIF expression in tumor and adjacent tissues; (B) Survival analysis of high and low MIF expression groups; (C) Expression of MIF in different tumor stages; (D) Differential expression analysis of tumors in high and low MIF expression groups; (E) Protein-protein interaction network of MIF co-expressed proteins in tumor tissues; (F) KEGG pathway enrichment analysis of MIF differential co-expressed genes.

Our previous studies established that MIF overexpression can facilitate the malignant transformation of AT2 cells^19^. As anticipated, the expression level of MIF in tumor tissues was significantly upregulated compared to healthy tissues (Figure 4A). Patients with driver gene-negative lung adenocarcinoma with high MIF expression had a poorer prognosis, and MIF expression levels were higher in patients with advanced disease (Figure 4B, C). To investigate the specific mechanisms underlying the influence of MIF on the development of driver gene-negative lung adenocarcinoma, differential expression analysis was carried out based on MIF expression levels in patients, leading to the identification of 906 differentially expressed genes, with the top 50 genes shown in Figure 4D. Additionally, 294 co-expressed genes of MIF were identified in the tumor tissues of driver gene-negative lung adenocarcinoma, and genes with high correlation were utilized to construct a co-expression network (Figure 4E). Intersecting the aforementioned differentially expressed genes with the co-expressed genes yielded 128 co-expressed genes with differential expression. KEGG enrichment analysis revealed that these genes were primarily enriched in signaling pathways such as Glycolysis/Gluconeogenesis, HIF-1 signaling pathway, Cell cycle, and Carbon metabolism (Figure 4F), implying that MIF primarily functions by influencing the glycolytic process in tumors, while the HIF-1α pathway is a key regulatory pathway.

### HIF-1α and MIF form a positive feedback regulatory axis to promote glycolysis and induce cellular malignant transformation

**Figure 5:**
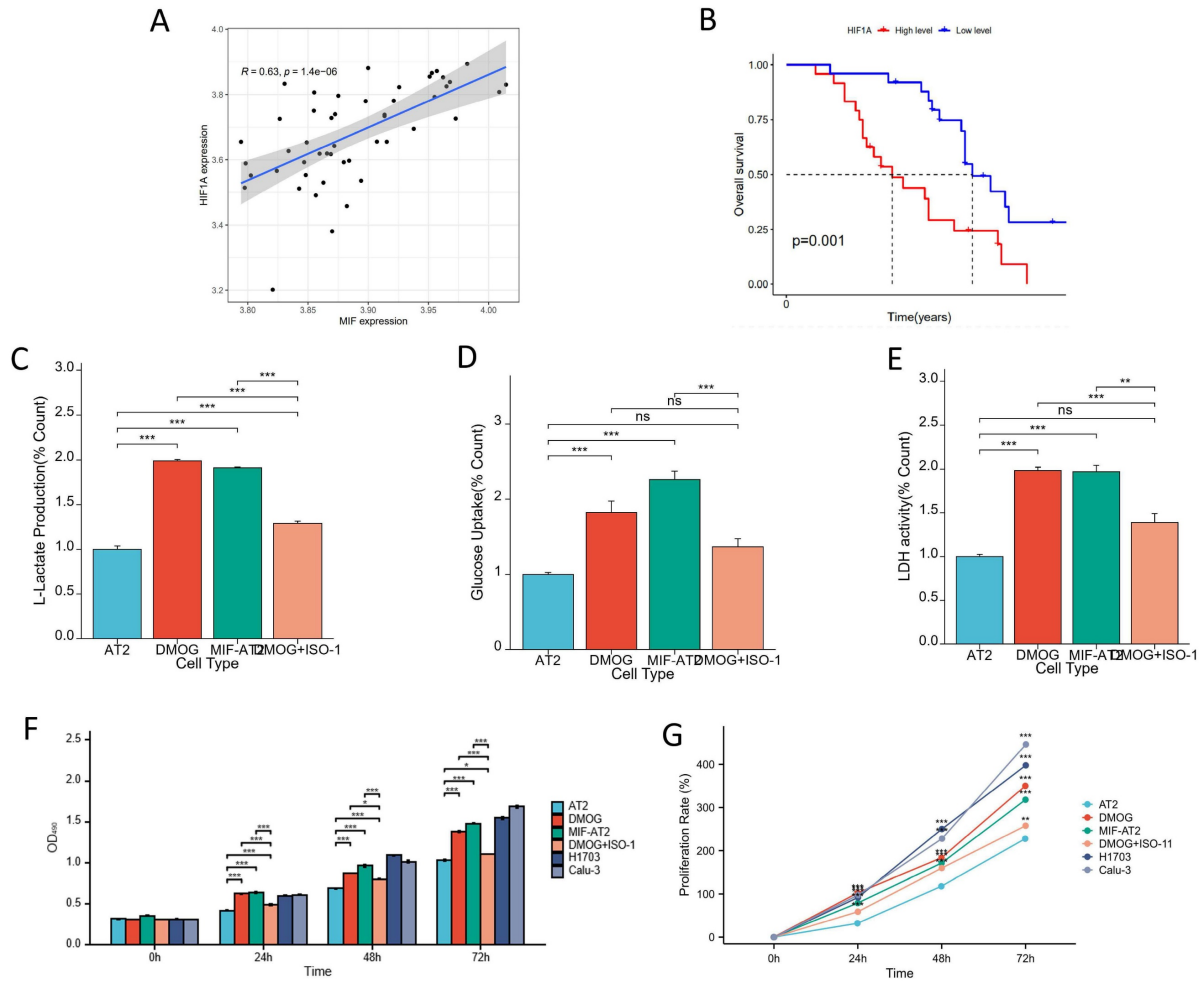
Correlation Analysis of MIF with HIF-1α and Cellular Experiments for Validation. (A) Correlation analysis of MIF and HIF-1α expression; (B) Survival analysis of high and low MIF expression groups; (C) Changes in L-Lactate production in AT2, DMOG, MIF-AT2 and DMOG+ISO-1; (D) Changes in glucose uptake rate in AT2, DMOG, MIF-AT2 and DMOG+ISO-1; (E) Changes in LDH activity in AT2, DMOG, MIF-AT2 and DMOG+ISO-1; (F) Cell proliferation in AT2, DMOG, MIF-AT2 and DMOG+ISO-1 calculated by OD490; (G) Cell proliferation curves of AT2, DMOG, MIF-AT2 and DMOG+ISO-1.

The correlation between the expression of MIF and HIF-1α was validated in driver gene-negative lung adenocarcinoma patients, and the result revealed a significant positive correlation between the expression levels of MIF and HIF-1α in tumor tissues (Figure 5A). Moreover, high expression of HIF-1α was associated with poor prognosis in patients with driver gene-negative LUAD (Figure 5B). To further corroborate the interaction between the two proteins, cell-based experiments were conducted, and the finding uncovered that MIF overexpression and HIF-1α activation (DMOG) both increased L-lactate levels in AT2 cells, whereas MIF inhibition (ISO-1) reversed the increase in L-lactate production in AT2 cells induced by HIF-1α activation (DMOG) (Figure 5C). Similar trends were observed in glucose uptake and LDH activity (Figures 5D and E), signaling that both MIF and HIF-1α significantly enhanced the glycolysis of AT2 cells, which was reversed by MIF inhibitors. However, regardless of MIF overexpression and HIF-1α activation, the glucose metabolism index levels of AT2 cells after treatment were still significantly lower than those of H1703 and Calu-3 lung cancer cell lines (Supplementary Figure 3A-C). Treatment of AT2 cells with the HIF-1α agonist DMOG significantly enhanced their proliferative capacity and this effect was also reversed by ISO-1 (Figures 5F and G). It was found that their proliferation rate was still significantly lower than that of lung cancer cell lines after 48 hours of upregulation of MIF or HIF-1α, and the proliferation rate of MIF+AT2 was higher than that of DMOG and closer to tumor cells (Supplementary Figure 3D). Overall, these results suggest that HIF-1α and MIF form a positive feedback regulatory axis that promotes glycolysis and drives cellular malignant transformation.

## Discussion

Driver gene-negative lung adenocarcinoma accounts for approximately 22%-55% of all lung adenocarcinoma cases and lacks effective treatment modalities^5^. Currently, targeted research on driver gene-negative lung adenocarcinoma is scarce, with the majority of studies investigating clinical and pathological characteristics and biomarkers for immunotherapy. Previous studies have reported that, in addition to PD-L1 and the tumor microenvironment, systemic inflammatory factors^20^, ^21^ and metabolic indicators^22^ can also predict the efficacy of immunotherapy in patients with driver gene-negative lung cancer.

Notably, metabolic reprogramming is a hallmark of malignant tumors. Previous studies have established that reprogramming of glucose metabolism is closely related to the development and progression of lung cancer^11^, ^13^. Our previous research revealed significant differential expression of glycolysis-related pathways in driver gene-negative lung adenocarcinoma tumors and adjacent tissues and that high-risk patients identified by radiomics likewise exhibited a significant increase in glucose metabolism^12^. Herein, bioinformatics analysis and statistical methods were performed to explore the gene expression profiles of 49 patients with driver gene-negative lung adenocarcinoma. Furthermore, GSEA enrichment analysis and LASSO regression were used to identify six characteristic genes (ANKZF1, GPR87, KIF2A, LCT, MIF, SDHC). These six characteristic genes have been proven in previous studies to promote the occurrence and development of various tumors by affecting glucose metabolism processes. ANKZF1, an ankyrin repeat and zinc finger domain-containing protein, is implicated in tumor progression and glycolysis across cancers. In colorectal cancer^23^ and hepatocellular carcinoma^24^, it was identified as an independent prognostic biomarker. GPR87 exhibits dual roles in tumor progression and glycolysis: it acts as an oncogene in melanoma by promoting glycolysis and suppressing immune responses through AKT/LDHA pathway activation^25^, while functioning as a tumor suppressor in prostate cancer by inhibiting glycolysis^26^. KIF2A is implicated in tumor progression and glycolysis, WAC-AS1 promotes glycolysis and proliferation by sponging miR-320d to upregulate KIF2A expression in hepatocellular carcinoma^27^. LCT is implicated in endometrial cancer progression through its association with glycolysis. High LCT expression correlates with aggressive tumor features and poorer survival^28^. SDHC, a subunit of succinate dehydrogenase (SDH), plays a dual role in tumorigenesis and glycolysis. Its dysfunction, such as epigenetic inactivation, drives metabolic reprogramming by suppressing mitochondrial respiration and enhancing glycolysis, thereby promoting tumor progression and metastasis in cancers^29^, ^30^. Meanwhile, single-cell sequencing was employed to conduct subpopulation analysis at the single-cell level in driver gene-negative lung adenocarcinoma, ultimately identifying MIF as a key gene involved in regulating glycolysis and playing a crucial role in regulating malignant cell transformation. Of note, the results of preliminary experiments validated our findings.

Macrophage migration inhibitory factor (MIF) is a multifunctional cytokine that plays a pivotal role in tumorigenesis, cancer progression, and inflammatory responses^31^, ^32^. It exerts various biological effects through various intracellular and extracellular signaling pathways^32^, including binding to receptors such as CD74 and CXCR, as well as the activation of Akt and NF-kB pathways^33^. In patients with lung cancer, MIF overexpression is associated with poor prognosis and is regarded as a candidate biomarker for non-small cell lung cancer^34^-^36^. Specifically, MIF overexpression directly enhances the growth and metastasis of lung cancer^20^, whereas its inhibition attenuates tumor progression by suppressing angiogenesis and metastasis^33^, ^37^. Importantly, MIF also plays a vital role in glucose metabolism. Indeed, it can influence glucose homeostasis by promoting insulin release while concurrently regulating glucose uptake, glycolysis, and insulin resistance in target cells. According to earlier studies, MIF overexpression significantly promotes the Warburg effect in H524 cells^38^, ^39^. This study aimed to explore the upstream and downstream pathways of the key gene MIF and identify potential regulatory mechanisms. By constructing a PPI co-expression network and performing KEGG pathway enrichment analysis, we discovered that the HIF-1α pathway is linked to the function of MIF.

The HIF-1α pathway modulates key biological processes such as glycolysis, cell proliferation, migration, and angiogenesis, which are essential for tumor survival and progression^20^. Noteworthily, HIF-1α is a known transcription factor for MIF^40^. The hypoxia-responsive element SNP rs17004038 in the MIF promoter region binds to HIF-1α, leading to the upregulation of MIF expression. Additionally, MIF can also affect HIF-1α activity. Earlier studies concluded that MIF can influence HIF-1α expression through the NF-κB pathway^41^ or regulate HIF-1 stability in a p53-dependent manner^42^. Besides, previous research indicated that MIF may promote the development of pancreatic cancer by forming a positive feedback loop with HIF-1α^42^. In pancreatic cancer cell lines, MIF stabilizes the structure of HIF-1α through CSN5, which in turn further up-regulates the expression of MIF. In the present study, the correlation between MIF and HIF-1α expression was validated, and cellular experiments were conducted to validate our hypothesis^43^. We observed that the upregulation of MIF or HIF-1α can promote the glycolysis level and cell proliferation ability of AT2 cells, but still lower than that of lung cancer cell lines. It is worth noting that the enhanced glucose metabolism and proliferation ability caused by the upregulation of HIF-1α can be reversed by MIF inhibitors. But currently, there is a lack of clear evidence of the interaction between these two molecules in lung cancer. We speculate that the presence of a MIF/HIF-1α positive feedback regulatory loop impacts the development of driver gene-negative lung adenocarcinoma through glycolysis mechanisms. We observed their co expression and cross-talk between pathways in tumor tissues, but lacked direct evidence on how they interact with each other. This inference warrants further experimental confirmation.

Nevertheless, some limitations of this study cannot be overlooked. This study is limited by reliance on bulk RNA-seq, which may mask cell-type-specific contributions to the MIF-HIF-1α axis. While single-cell analysis identified AT2 cells as key drivers, the sample size restricts broader conclusions. Future work will integrate spatial transcriptomics and expand single-cell profiling to validate cell-state dynamics. Mechanistically, this study exclusively explored the selected key genes at the cellular level, direct interactions between MIF and HIF-1α remain unproven. We are addressing this gap through Co-IP/mass spectrometry and HIF-1α ChIP-seq to define their molecular interplay. These limitations underscore the need for deeper mechanistic interrogation and multi-omics integration to fully harness the therapeutic potential of the MIF-HIF-1α axis. Notably, our team has recently completed relatively in-depth experiments and more comprehensive methylation omics analysis for a subset of driver gene-negative LUAD samples^44^. We plan to perform integrative analyses of bulk RNA-seq, single-cell transcriptomics, and DNA methylation data to identify epigenetic regulators of the MIF-HIF-1α axis and their clinical implications.

## Conclusion

This study investigated patients with driver gene-negative LUAD, and multi-omics analysis revealed the instrumental role of the glycolysis pathway in tumorigenesis. Moreover, MIF was identified as a key gene affecting glycolysis and the malignant transformation of tumors. These findings collectively suggest that MIF may form a positive feedback regulatory axis with HIF-1α, promoting glycolysis and the malignant phenotype in tumor cells. These insights provide potential targets for the treatment of driver gene-negative LUAD. Despite limitations in sample size and study scope, the results offer valuable directions for future research and the development of effective treatment strategies.

## Supplementary Material

Supplementary figures and tables.
