# Supplementary material for: Exploring the Glycolytic Mechanisms in “Driver Gene-Negative” Lung Adenocarcinoma (LUAD): A Single-Cell RNA Sequencing Approach to Identify the MIF-HIF-1α Axis
Source: J Cancer. 2025 Oct 10;16(14):4233–44. doi: 10.7150/jca.119149 (PMC12595263; doi:10.7150/jca.119149)
Supplement: Supplementary file 1 — Supplementary figures and tables. [file jcav16p4233s1.pdf]

Supplementary Material

Supplementary figure 1

Supplementary figure 2

Supplementary figure 3

Supplementary Table 1

Supplementary Table 2

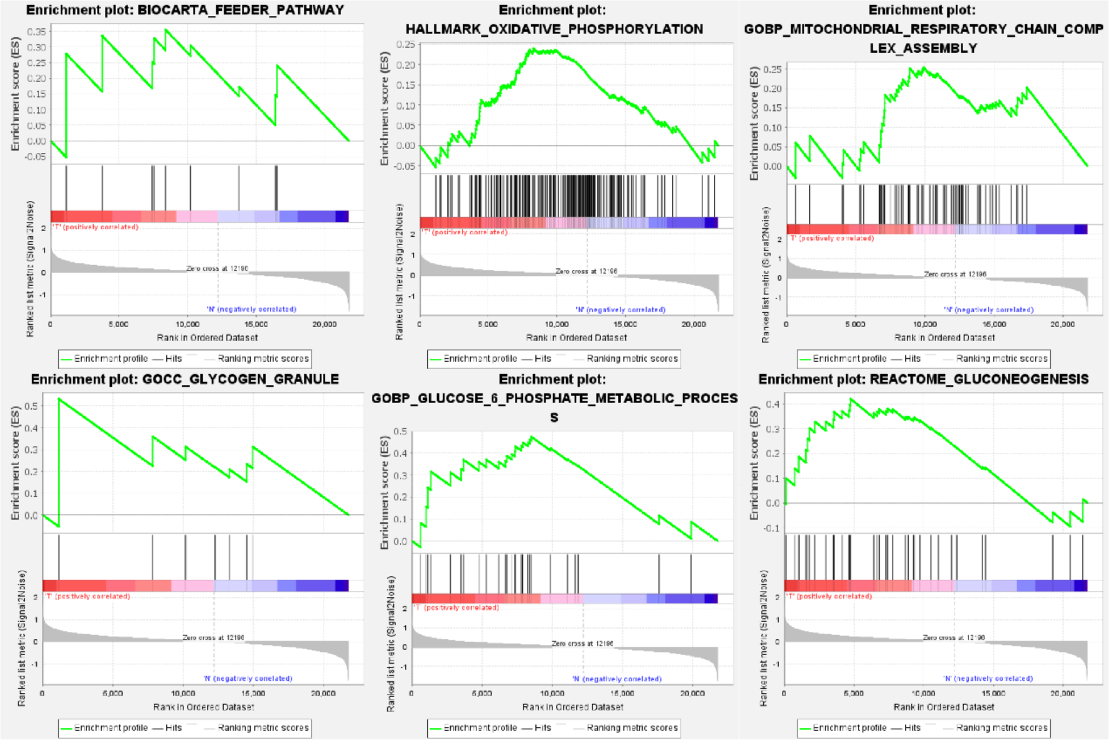

Supplementary Figure 1: Enrichment of Glycolytic Pathways in Driver Gene-Negative Lung Adenocarcinoma and Adjacent Normal Tissues.

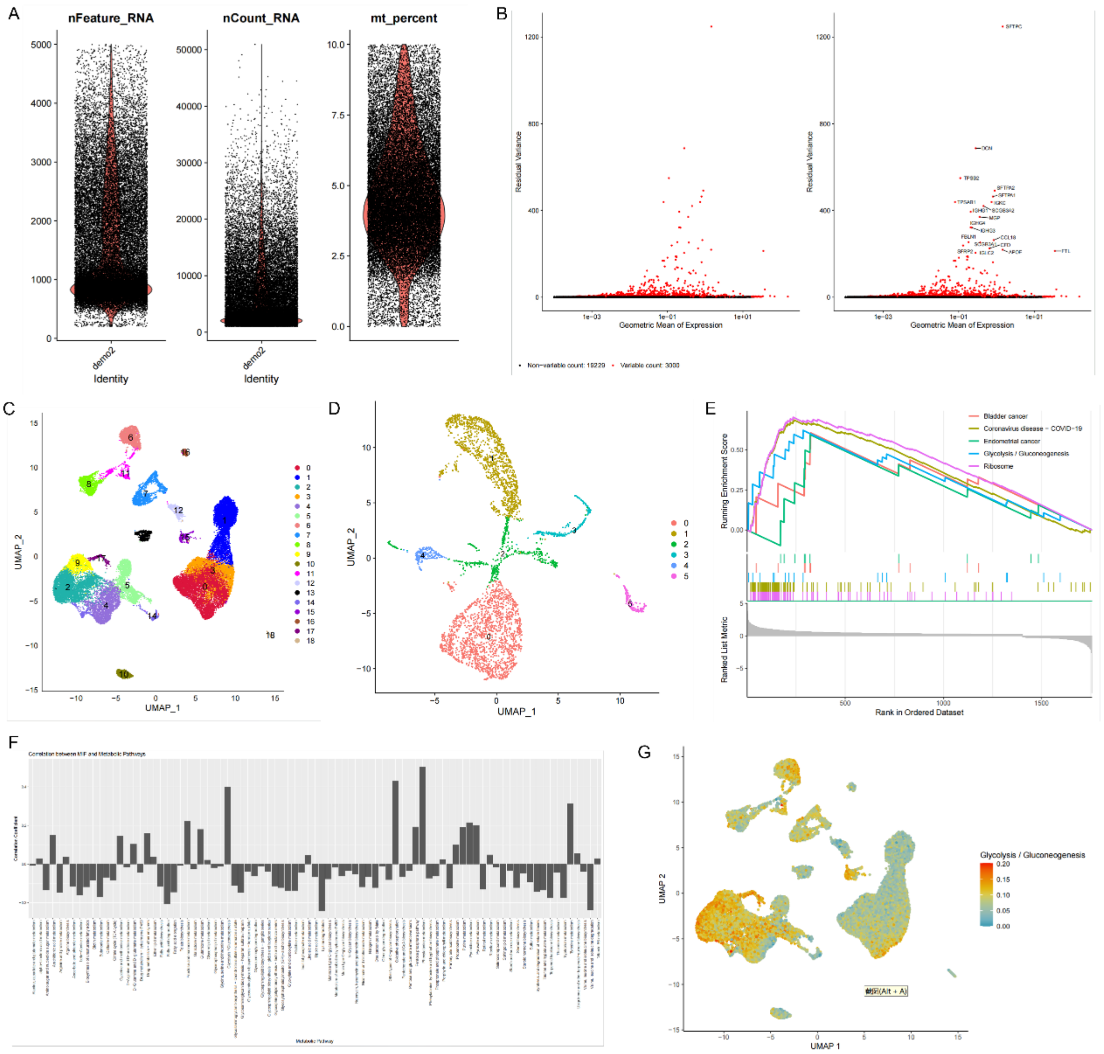

Supplementary Figure 2: Results of Single-Cell Analysis Quality Control, Clustering, and Pathway Enrichment. (A) RNA quantity and mitochondrial RNA characteristics after single-cell data quality control; (B) Identification and annotation of highly variable genes; (C) Clustering of single cells from driver gene-negative lung adenocarcinoma patients; (D) GSEA enrichment results of MIF+AT2 cells relative to AT2 cells; (E) Clustering of epithelial cells; (F) Correlation analysis of MIF with metabolic pathways in MIF+AT2 cells; (G) Enrichment of glycolytic pathways in driver gene-negative lung adenocarcinoma tumor and normal tissues.

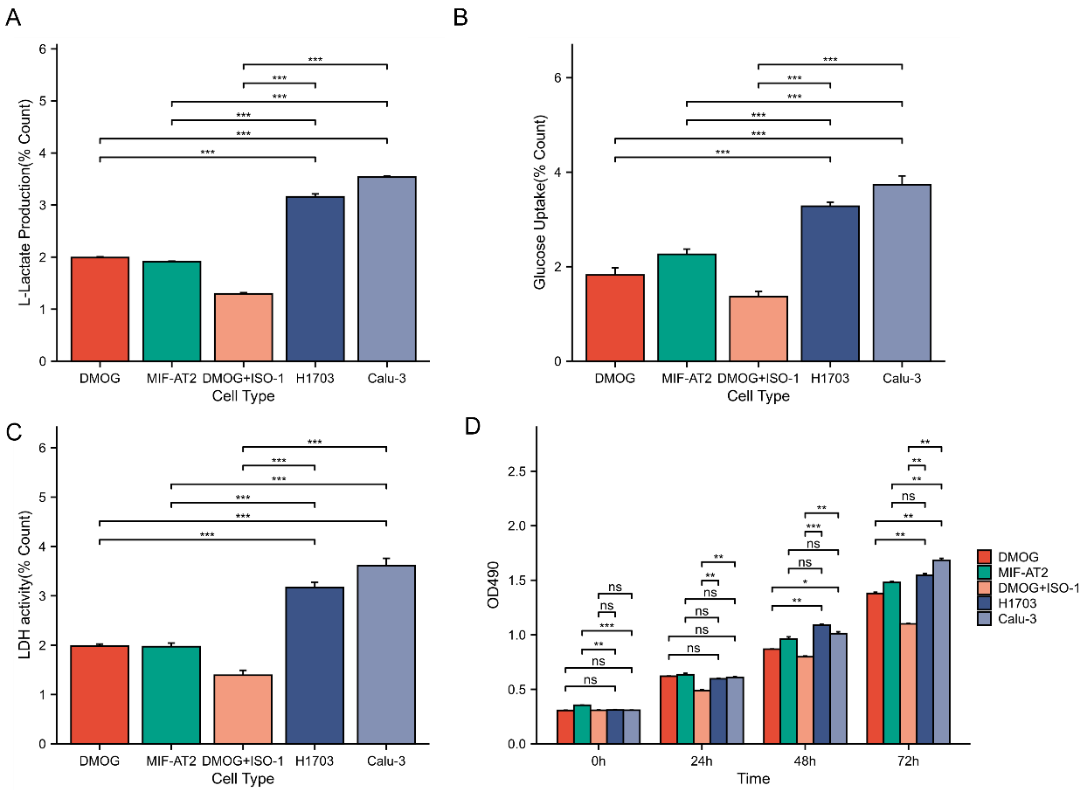

Supplementary Figure 3: Comparison of MIF or HIF-1α upregulation treatment with tumor cell lines; (A) Changes in L-Lactate production in DMOG, MIF-AT2, DMOG+ISO-1, H1703 and Calu-3; (B) Changes in glucose uptake rate in DMOG, MIF-AT2, DMOG+ISO-1, H1703 and Calu-3; (C) Changes in LDH activity in DMOG, MIF-AT2, DMOG+ISO-1, H1703 and Calu-3; (D) Cell proliferation in DMOG, MIF-AT2, DMOG+ISO-1, H1703 and Calu-3 calculated by OD490.

Gene	conMean	treatMean	logFC	pValue
CENPA	2.201587949	5.558139114	1.336057466	1.47E-10
SLC25A10	1.628318173	3.359737102	1.044965717	1.85E-13
KIF20A	2.815778429	5.725869753	1.02396104	3.18E-18
DSC2	2.277439827	4.414522	0.954844296	4.63E-10
LCT	1.77782159	3.247124682	0.869052229	0.001613794
SOX9	4.600134629	8.364096098	0.862533554	4.14E-20
HS6ST2	4.443061973	7.458845861	0.747398149	9.74E-15
B3GNT3	5.539661747	9.220603016	0.735063217	4.85E-16
ALDOB	1.802393831	2.9418767	0.706822501	3.44E-05
SPAG4	3.982381939	6.4262849	0.69035335	1.67E-15
GALE	3.528200792	5.483861178	0.636259382	1.15E-14
MIOX	3.260491873	4.801030727	0.558254545	6.38E-21
DEPDC1	3.4433306	4.936981	0.51982439	1.10E-12
BIK	5.26419021	7.540131239	0.518378016	4.62E-17
LDHC	4.376528812	6.253673453	0.51491682	4.40E-14
HMMR	5.446673969	7.372611367	0.436800198	2.87E-13
AURKA	4.913517233	6.647860778	0.436134053	1.24E-14
CDK1	6.076191588	8.2162943	0.435320499	4.89E-18
G6PD	2.982607143	4.004769031	0.425145076	6.00E-07
VEGFA	10.28558066	13.76139053	0.420003014	7.85E-29
KDELR3	7.139780808	9.490840235	0.410656032	2.95E-23
GPR87	4.659729696	6.013069616	0.367855393	0.009084416
GMPPA	3.279790296	4.227941827	0.366351952	2.76E-08
B4GALT1	6.096498824	7.763037547	0.348640314	1.12E-09
TPBG	7.770138451	9.69737202	0.319653526	2.97E-19
CHPF2	4.284354649	5.344900243	0.319085114	2.78E-09
B4GALT7	4.106053831	5.078304796	0.306594446	2.01E-11
ELF3	7.404371643	9.139108206	0.303676085	4.90E-09
ANKZF1	4.876964908	5.942581451	0.285106181	3.62E-07
IGFBP3	9.193392165	11.17840611	0.282045306	2.44E-10
DPYSL4	5.607768394	6.806122824	0.279406421	6.87E-11
PFKFB1	1.441812606	1.747171722	0.277137744	0.000828711
PLOD2	8.004164539	9.698957635	0.277078882	6.50E-10
AK4	7.026189361	8.471081465	0.269803705	4.43E-09
PMM2	6.807202831	8.203770516	0.269225038	1.01E-09
SDC1	10.67756287	12.85982068	0.268288134	6.37E-24
NT5E	6.042575859	7.263542133	0.265509583	5.64E-05
ENO1	9.586965276	11.46289761	0.257825665	4.04E-16
CLN6	4.724127447	5.646974961	0.257430348	2.50E-09
AKR1A1	5.640147718	6.731464788	0.255187526	5.22E-12
PPFIA4	5.325439878	6.352438476	0.254409802	7.31E-05
B4GALT2	6.52208242	7.758503471	0.250445727	4.89E-12
GNPDA1	5.220776737	6.197241682	0.247361768	8.12E-08

FUT8	7.904717606	9.350853827	0.24238418	4.40E-18
PC	7.07318391	8.360515751	0.24123217	4.03E-14
AGRN	6.443085555	7.607370682	0.239646153	1.12E-09
COL5A1	6.30757269	7.444191224	0.239030188	1.40E-10
CHST4	4.642969798	5.451693386	0.231656528	3.93E-05
PKP2	5.924324943	6.942303322	0.228763628	0.001215971
PLOD1	3.19058049	3.736277367	0.22778263	4.19E-06
PSMC4	6.816916076	7.891728278	0.211222061	1.16E-06
IER3	7.65479948	8.851629863	0.209578539	3.62E-07
TGFA	6.005448816	6.921154616	0.204740665	2.51E-08
GPC1	6.769682343	7.790424314	0.202613769	5.78E-07
P4HA1	9.77533552	11.23502416	0.200785101	1.80E-11
NDST3	1.625464384	1.861461898	0.195584141	0.010534692
LDHA	10.84490105	12.34959617	0.187446977	1.40E-15
ZNF292	5.985287133	6.794961896	0.183045001	5.73E-11
ALDOA	9.984347643	11.33469765	0.183005835	1.32E-07
CHST6	7.721897596	8.754026698	0.180991362	2.90E-08
CLDN3	10.69616639	12.11962087	0.180250755	3.04E-08
PDK3	5.508092178	6.224482855	0.176401277	3.93E-05
VCAN	9.558354816	10.7955199	0.175598495	6.43E-08
ALG1	7.350182457	8.273453335	0.170709572	1.44E-19
MED24	5.082963669	5.714359759	0.168921948	5.66E-06
PFKP	8.747139959	9.811956616	0.165729477	1.47E-08
GMPPB	4.950507084	5.55084471	0.165131024	0.000250099
CLDN9	3.124273057	3.502958049	0.165053162	0.000128772
SLC37A4	6.911718359	7.731927016	0.161783588	6.98E-14
ME1	6.242681224	6.976504035	0.160338477	0.000117346
IDH1	9.704220571	10.83798261	0.15941199	9.50E-12
GFPT1	8.771487308	9.79415259	0.159099184	7.46E-18
CHPF	8.9025558	9.915902839	0.155524562	1.10E-16
LHPP	7.004815157	7.791530141	0.153559699	2.10E-10
SRD5A3	6.940776408	7.705077596	0.150712431	0.001095136
EGLN3	7.601912024	8.42308682	0.147986707	0.000589072
CASP6	7.20403231	7.972282188	0.146188125	2.03E-08
FKBP4	6.514207545	7.177509104	0.13989357	1.17E-05
HDLBP	8.803897984	9.696976204	0.139392516	1.11E-07
SDHC	8.293248673	9.123910927	0.13771501	3.37E-16
PYGB	8.855219469	9.705792	0.132317879	2.22E-06
PGK1	12.13397127	13.28298754	0.130527867	1.07E-08
GOT1	8.518843967	9.314658888	0.128845271	3.25E-09
PGAM1	8.702195618	9.505566386	0.127393144	4.04E-08
PAXIP1	7.628627394	8.324709527	0.125976434	1.10E-13
MERTK	6.35908901	6.926484943	0.123303295	3.85E-08
ME2	7.474414902	8.1275929	0.120867492	1.17E-06

RPE	7.982284922	8.639096604	0.114078681	1.56E-11
GALK1	3.950071159	4.27504069	0.114059508	0.008518943
ADORA2B	7.859307351	8.503443457	0.113645005	0.001857665
MIF	12.77891545	13.82598071	0.113616419	8.02E-16
COPB2	11.92950953	12.81233689	0.102998909	1.72E-12
B4GALT4	8.474471133	9.085682098	0.100471491	0.002290572
GLCE	7.249783839	7.766982584	0.099416251	3.74E-06
GPC4	9.202426663	9.858664594	0.099377892	1.09E-05
GYS1	7.6111323	8.149334327	0.098571121	0.000294471
STMN1	6.758440429	7.216201908	0.094549336	0.001280852
NSDHL	9.595542378	10.24350766	0.094273558	3.12E-07
ARTN	4.873984545	5.202728729	0.09416681	0.006852496
POLR3K	7.694920863	8.194006902	0.090662618	7.60E-06
CTH	7.031157061	7.483848196	0.090018173	0.000458202
RBCK1	5.521807186	5.876489304	0.089814015	0.011451902
ABCB6	10.15492887	10.80357717	0.089328948	1.24E-05
COG2	8.924185867	9.484385367	0.087833722	1.29E-11
FAM162A	11.63906767	12.36276478	0.087025921	2.69E-07
PGLS	10.56801788	11.20813486	0.084841407	5.52E-07
SLC25A13	8.368733953	8.870996408	0.084086776	2.56E-07
SLC35A3	6.664850751	7.060507435	0.083199304	0.011691893
CHST2	9.077546133	9.613118602	0.082702176	0.025677797
SLC16A3	11.99474009	12.68506914	0.080729486	1.39E-05
DDIT4	10.90201023	11.51092329	0.078409376	0.01922319
BPNT1	8.038547653	8.486850853	0.078294455	8.57E-05
MDH2	10.82710885	11.41308113	0.076040269	3.26E-06
TPI1	11.2463674	11.8380191	0.073968607	0.001011838
MPI	8.678138143	9.120202337	0.07168028	2.40E-05
KIF2A	7.755412416	8.133774314	0.068721459	0.000409146
TXN	12.57296463	13.14303717	0.063973832	0.00017082
ARPP19	8.614841918	9.004855369	0.063878781	0.003372019
PGM2	8.967038816	9.337796767	0.058450547	0.020072415
TALDO1	12.699272	13.20374513	0.056201401	5.52E-06
TPST1	10.1288969	10.51317711	0.053721658	0.002992774
IL13RA1	10.21473202	10.56911667	0.049203451	0.000605566
SOD1	13.41347917	13.82326233	0.043414645	2.07E-06
HAX1	9.534492255	9.810348061	0.041148211	0.034229352
ECD	10.17711108	10.41960633	0.033972681	0.001348883
GOT2	11.87277273	12.07675591	0.024576069	0.021938113
PHKA2	9.634727786	9.425210939	-0.031718999	0.031327668
HSPA5	9.814500296	9.595947204	-0.032489595	0.047438959
GNE	9.70815999	9.429051051	-0.0420853	0.001877696
PPP2CB	10.65573699	10.1658807	-0.067895173	0.000648411
CYB5A	13.19740554	12.4670342	-0.082136038	0.000817277

CD44	13.63811168	12.78145472	-0.093591858	1.57E-10
ANG	8.743697857	8.127910335	-0.105359062	0.000757484
NANP	4.690772329	4.345181698	-0.110408974	0.001038936
GLRX	12.63320981	11.62063798	-0.120531965	1.72E-10
ALDH7A1	8.000524286	7.267373808	-0.13866043	0.000242826
IRS2	9.534574429	8.658702853	-0.139017633	2.80E-07
DCN	12.29746364	11.08131229	-0.150232049	5.59E-08
LHX9	3.696090398	3.226735449	-0.195924742	0.005007076
SDC2	6.210690108	5.145358322	-0.271482034	9.03E-10
CACNA1H	9.254325908	7.49401172	-0.304389673	1.01E-09
TKTL1	6.066209733	4.866936429	-0.317781448	7.53E-14
GPC3	10.77940301	8.377526594	-0.363681015	2.06E-18
FBP2	4.374283512	2.138631555	-1.032358774	4.42E-13

Supplementary Table 1 Differential expression analysis of glycolysis-related genes between the tumor and adjacent control groups

Gene	Coef
ANKZF1	-0.046220784
GPR87	0.122594146
KIF2A	0.179754057
LCT	-0.049561258
MIF	0.134607422
SDHC	0.296448982

Supplementary Table 2 The regression coefficients for each gene in model
